# Supplementary material for: Association between Childhood Overweight and Altered Concentrations of Circulating Amino Acids
Source: Nutrients. 2024 Jun 12;16(12):1843. doi: 10.3390/nu16121843 (PMC11206240; doi:10.3390/nu16121843)
Supplement: Supplementary file 1 [file nutrients-16-01843-s001.zip › nutrients-3050227-supplementary.pdf]

Supplementary Table S1. List of proteogenic and amino acids related

Proteogenic Amino acids		Aminoacids related	
**Ala**	Alanine	**alpha-AAA**	α-Aminoadipic acid
**Arg**	Arginine	**1-Met-His**	1-Methylhistidine
**Asn**	Asparagine	**3-Met-His**	3-Methylhistidine
**Asp**	Aspartic Acid	**5-AVA**	5-Aminovaleric acid
**Cys**	Cysteine	**AABA**	α-Aminobutyric acid
**Gln**	Glutamine	**Ac-Orn**	Acetylornithine
**Glu**	Glutamic Acid	**ADMA**	Asymmetric dimethylarginine
**Gly**	Glycine	**Anserine**	Anserine
**His**	Histidine	**BABA**	β-Aminobutyric acid
**Iso**	Isoleucine	**Betaine**	Betaine
**Leu**	Leucine	**c4-OH-Pro**	cis-4-Hydroxyproline
**Lys**	Lysine	**Carnosine**	Carnosine
**Met**	Methionine	**Cit**	Citrulline
**Phe**	Phenylalanine	**Creatinine**	Creatinine
**Pro**	proline	**Cystine**	Cystine
**Ser**	Serine	**DOPA**	Dihydroxyphenylalanine
**Thr**	Treonine	**HArg**	Homoarginine
**Trp**	Tryptophan	**HCys**	Homocysteine
**Tyr**	Tyrosine	**Kynurenine**	Kynurenine
**Val**	Valine	**Met-SO**	Methionine sulfoxide
		**Nitro-Tyr**	Nitrotyrosine
		**Orn**	Ornithine
		**PAG**	Phenylacetylglycine
		**PheAlaBetaine**	Phenylalanine betaine
		**ProBetaine**	Proline betaine
		**Sarcosine**	Sarcosine
		**SDMA**	Symmetric dimethylarginine
		**t4-OH-Pro**	trans-4-Hydroxyproline
		**Taurine**	Taurine
		**TrpBetaine**	Tryptophan betaine

Supplementary figure S1 (A,B)

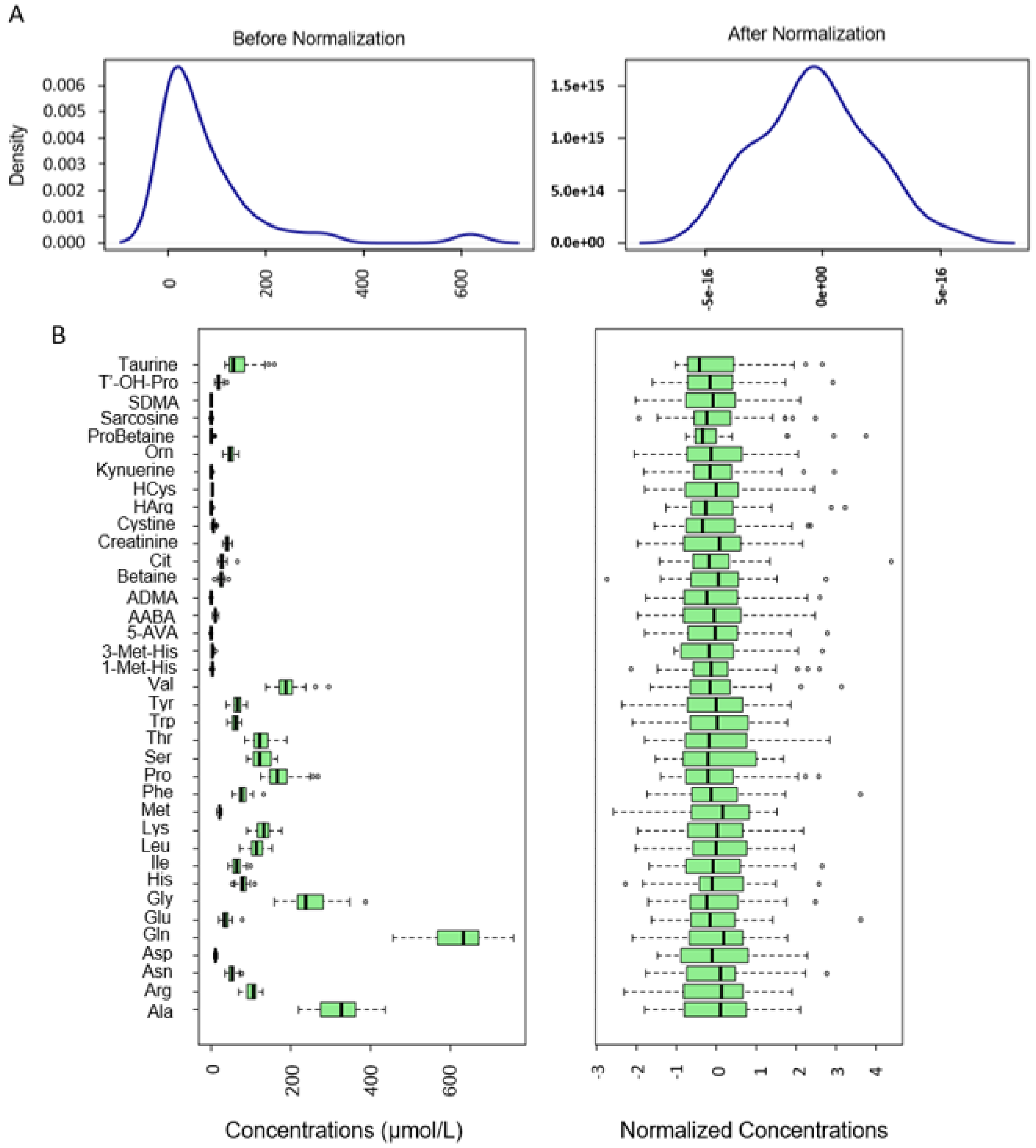

Supplementary figure S1 (C)

C

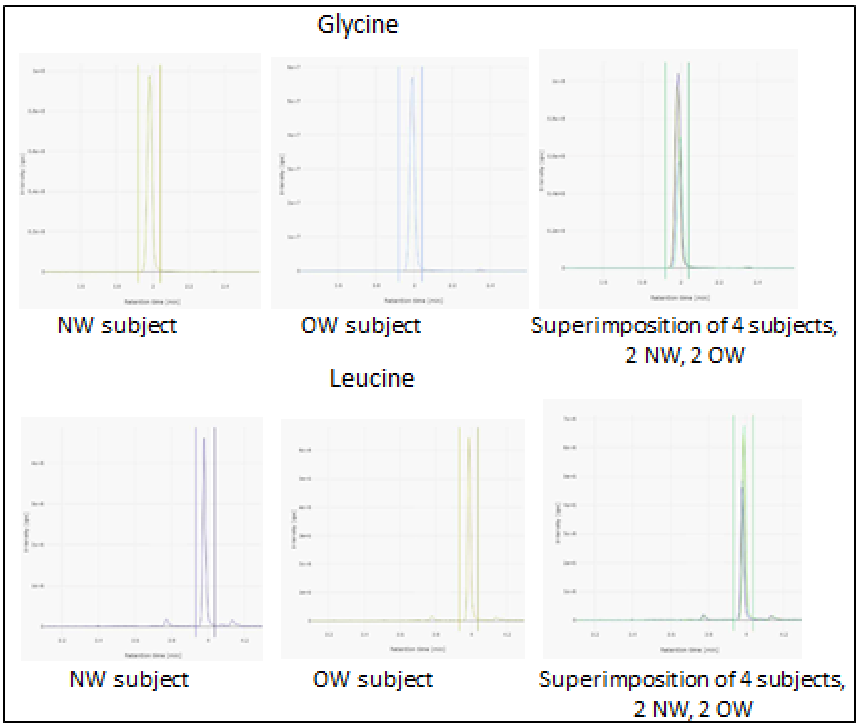

Supplementary figure S2

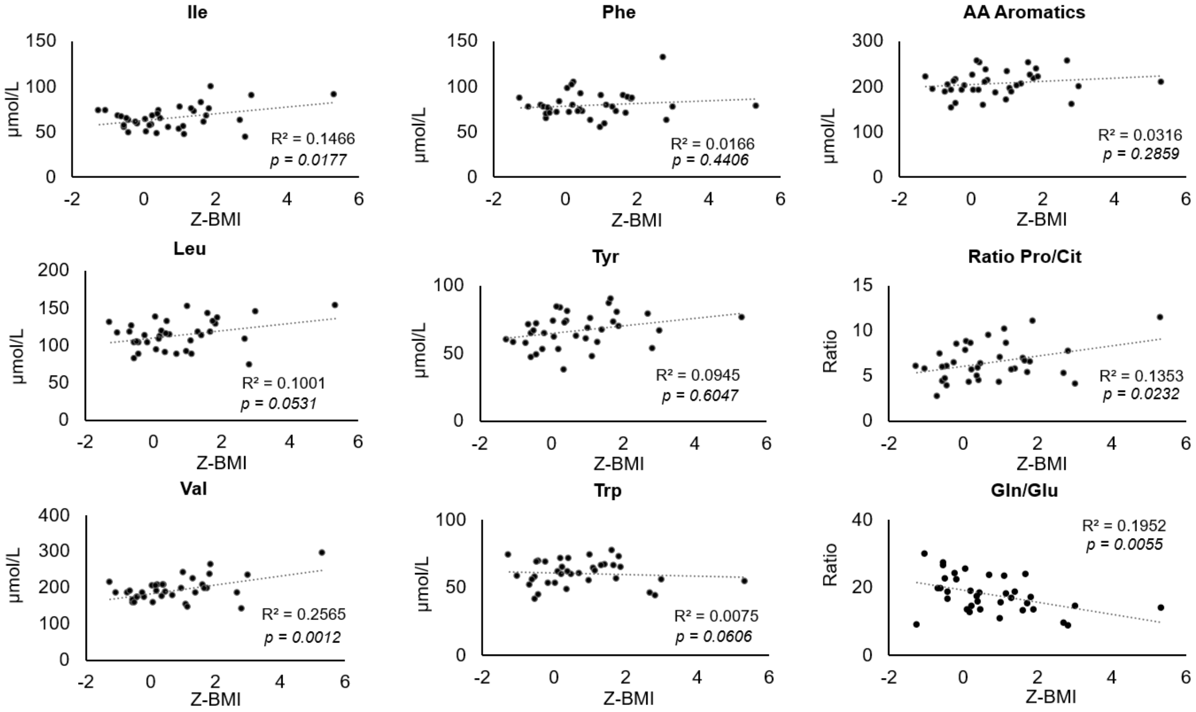

Supplementary figure S3

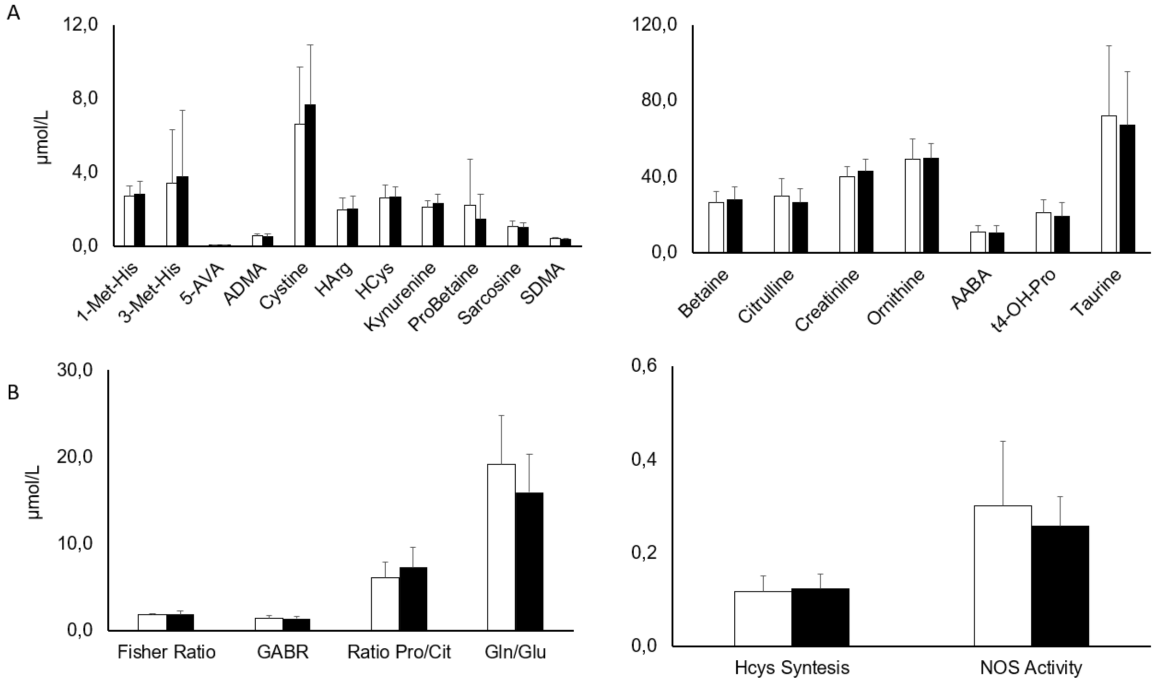

Supplementary figures legends

Supplementary Figure S1. Data Normalization prior to metabolomics analysis. A. Density distribution (upper graph) and metabolite concentrations (lower graph) prior to normalization. **B.** Density distribution (upper graph) after auto scaling (mean-centered and divided by the standard deviation of each variable) and normalized metabolite concentrations (lower graph). **C.** Representative LC-MS/MS chromatographic separations of Glycine (upper part) and Leucine (Lower part). A separation from a NW subject (left), and OW subject (center), and overlapping peaks from 2 NW and 2 OW subjects (right) are shown.

Supplementary Figure S2: correlations between plasma AA and ratios of amino acids concentrations and BMI z-Score in children aged 7-12. Ile: isoleucine, Leu: leucine, Val: valine, Phe: phenylalanine, Tyr: tyrosine, Trp: Tryptophan, AAA: aromatics amino acids (Phe+ Tyr+ Trp), Ratio Pro/Cit: proline/ citrulline, Gln/Glu: glutamine/glutamic acid. R^2 correlation coefficients and significance are reported on each graph.

Supplementary Figure S3: A. Amino acids related concentrations and amino acids ratios in normal weight and overweight children aged 7-12. AABA: α-Aminobutyric acid; ADMA: Asymmetric dimethylarginine; 5-AVA: 5-Aminovaleric acid; Cit: Citrulline; Harg: Homoarginine; HCys: Homocysteine; t4-OH-Pro: trans-4-Hydroxyproline; 1-Met-His: 1-Methylhistidine; 3-Met-His: 3-Methylhistidine; Orn: Ornithine; ProBetaine: Proline betaine; SDMA: Symmetric dimethylarginine. **B.** Fisher ratio: BCAA/AAA; GABR: Arg/(Orn+Cit); Hcys syntesis: Hcys/ Met; Ratio Pro/Cit: Proline/Citrulline; NOS activity: Citrulline/arginine; Gln/Glu: Glutamine/Glutamic acid. Data represent means and standard deviations. For all t-test comparisons, significance was >0.05.
